# Predicting Readmission Charges Billed by Hospitals: Machine Learning Approach

**DOI:** 10.2196/37578

**Published:** 2022-08-30

**Authors:** Deepika Gopukumar, Abhijeet Ghoshal, Huimin Zhao

**Affiliations:** 1 Department of Health and Clinical Outcomes Research School of Medicine Saint Louis University St.Louis, MO United States; 2 Department of Business Administration Gies College of Business University of Illinois Urbana-Champaign Champaign, IL United States; 3 Sheldon B Lubar College of Business University of Wisconsin-Milwaukee Milwaukee, WI United States

**Keywords:** readmission charges, readmission analytics, predictive models, machine learning, readmissions, predictive analytics

## Abstract

**Background:**

The Centers for Medicare and Medicaid Services projects that health care costs will continue to grow over the next few years. Rising readmission costs contribute significantly to increasing health care costs. Multiple areas of health care, including readmissions, have benefited from the application of various machine learning algorithms in several ways.

**Objective:**

We aimed to identify suitable models for predicting readmission charges billed by hospitals. Our literature review revealed that this application of machine learning is underexplored. We used various predictive methods, ranging from glass-box models (such as regularization techniques) to black-box models (such as deep learning–based models).

**Methods:**

We defined readmissions as readmission with the same major diagnostic category (RSDC) and all-cause readmission category (RADC). For these readmission categories, 576,701 and 1,091,580 individuals, respectively, were identified from the Nationwide Readmission Database of the Healthcare Cost and Utilization Project by the Agency for Healthcare Research and Quality for 2013. Linear regression, lasso regression, elastic net, ridge regression, eXtreme gradient boosting (XGBoost), and a deep learning model based on multilayer perceptron (MLP) were the 6 machine learning algorithms we tested for RSDC and RADC through 10-fold cross-validation.

**Results:**

Our preliminary analysis using a data-driven approach revealed that within RADC, the subsequent readmission charge billed per patient was higher than the previous charge for 541,090 individuals, and this number was 319,233 for RSDC. The top 3 major diagnostic categories (MDCs) for such instances were the same for RADC and RSDC. The average readmission charge billed was higher than the previous charge for 21 of the MDCs in the case of RSDC, whereas it was only for 13 of the MDCs in RADC. We recommend XGBoost and the deep learning model based on MLP for predicting readmission charges. The following performance metrics were obtained for XGBoost: (1) RADC (mean absolute percentage error [MAPE]=3.121%; root mean squared error [RMSE]=0.414; mean absolute error [MAE]=0.317; root relative squared error [RRSE]=0.410; relative absolute error [RAE]=0.399; normalized RMSE [NRMSE]=0.040; mean absolute deviation [MAD]=0.031) and (2) RSDC (MAPE=3.171%; RMSE=0.421; MAE=0.321; RRSE=0.407; RAE=0.393; NRMSE=0.041; MAD=0.031). The performance obtained for MLP-based deep neural networks are as follows: (1) RADC (MAPE=3.103%; RMSE=0.413; MAE=0.316; RRSE=0.410; RAE=0.397; NRMSE=0.040; MAD=0.031) and (2) RSDC (MAPE=3.202%; RMSE=0.427; MAE=0.326; RRSE=0.413; RAE=0.399; NRMSE=0.041; MAD=0.032). Repeated measures ANOVA revealed that the mean RMSE differed significantly across models with *P*<.001. Post hoc tests using the Bonferroni correction method indicated that the mean RMSE of the deep learning/XGBoost models was statistically significantly (*P*<.001) lower than that of all other models, namely linear regression/elastic net/lasso/ridge regression.

**Conclusions:**

Models built using XGBoost and MLP are suitable for predicting readmission charges billed by hospitals. The MDCs allow models to accurately predict hospital readmission charges.

## Introduction

### Background

Electronic health records (EHRs) are now widely adopted by hospitals. EHR adoption has almost doubled since 2008, one of the reasons being the implementation of the government-related mandate as part of the American Recovery and Reinvestment Act of 2009 [1,2]. Even with the implementation of technological innovations like EHRs and various reforms for funding health care initiatives, health care costs have continued to increase. As per the recent National Health Expenditure Fact Sheet provided by the Centers for Medicare and Medicaid Services (CMS), the national health expenditure has grown 9.7% by the end of 2020, totaling US $4.1 trillion (approximately 19.7% of the Gross Domestic Product). On average, the United States of America spends over US $10,000 per resident per year toward health care. It is considerably higher than that in other countries included in the Organization for Economic Co-operation and Development, where the average cost is only US $4000 per person after adjusting for purchasing power [3].

Readmissions have been a significant contributor to rising health care costs. The hospital cost associated with 30-day all-cause readmissions was approximately US $41.3 billion for 2011 [4]. Even before the pandemic, annual hospital readmission costs were approximately US $26 billion for Medicare alone [5]. The pandemic caused a further increase in readmission costs [6]. Being expensive at the individual level, readmission is often postponed by patients until their health severely degenerates, leading to further increases in readmission costs, and these in turn contribute to the rapidly rising health care costs.

As a result, it is important for hospitals to plan for potential readmissions and associated costs. Although past research has primarily focused on predicting the probability of readmissions, the cost of readmissions is understudied, which is an important element in the financial planning done by hospitals as well as various concerned governmental agencies. As our task is to predict future hospital readmission charges, we take cues from existing literature on predictive analytics that have been applied and found beneficial in multiple areas of health care, such as risk analysis, disease diagnosis, disease progression, and preventive care [7-12]. Thus, we expect that predicting hospital readmission charges would help hospital policymakers plan for the upcoming expenditures. Hospitals can use these predictions to design policies based on the costs borne by individual patients.

According to the CMS, readmission is defined as an admission to a hospital within 30 days of discharge [13]. It could be from the same or another hospital, irrespective of the cause of readmission. However, readmission charges can be expected to vary significantly across major diagnostic categories (MDCs). To better control such variations and develop effective prediction models, we consider predicting the charges based on MDCs in this study, which is a novel aspect of our research. To the best of our knowledge, this aspect has not been explored in the past. We compare the predictions with the case when all diagnostic categories are pooled to predict readmission charges. Accordingly, we deploy the term readmission in two ways: readmission with the same major diagnostic category (RSDC) and all-cause readmission category (RADC). RSDC is defined as an admission to a hospital (same or another hospital) within 30 days of discharge with the cause of readmission being the same as the previous admission. In this context, the “cause of readmission” is based on the major diagnostic category (MDC). RADC is defined as an admission to a hospital within 30 days of readmission, irrespective of the cause of readmission.

### Objective

The hospital charges for readmitted individuals can vary based on different services (such as procedures, labs, X-rays, and scans) used. Predicting these charges would be beneficial for financial planning by hospitals. Existing studies mainly focus on predicting either readmission probabilities or general health care costs. To date, no thorough research on suitable machine learning models exists for predicting hospital readmission charges. An exception is a study focusing on predicting readmission costs (not charges) [14]; however, it also does not include modern approaches, such as deep learning and regularization-based techniques. Our objective is to consider and compare traditional and modern predictive techniques to identify a suitable approach for predicting readmission charges.

Before building predictive models for RSDC and RADC, we also conducted preliminary analyses. First, for understanding the contribution of readmissions to the rising health care costs based on different criteria for readmissions (ie, RSDC and RADC), we determined the variation in the percentage of individuals contributing to hospital charges in our research context. Next, we analyzed whether readmissions varied across MDCs based on RSDC and RADC. As readmission policies vary across countries, we analyzed different readmission criteria for MDCs. Then, we determined whether the readmission charges changed significantly compared to the previous admission charges for RSDC and RADC. Finally, we strived to build models for predicting readmission charges billed by hospitals for RSDC and RADC.

### Prior Work

The literature on applications of predictive methods for health care outcomes is vast. We focus on discussing works that directly relate to our study and context. Numerous machine learning–based approaches have been applied to predict readmissions and health care costs. For the sake of brevity, we list them succinctly in Table 1.

**Table 1:** Models used in prior studies.

Prediction area	Contexts and models used
Readmissions	All-cause: Artificial neural network (Jamei et al [15]); Bayesian network (Cai et al [16]); bidirectional encoder representation from transformers (Huang et al [17]); convolutional neural network (Wang et al [18]); Cox regression model (Yu et al [19]); decision trees (Sushmita et al [14] and Shadmi et al [20]); generalized boosting model (Sushmita et al [14]); multilayer perceptron (Wang et al [18]); multiple logistic regression (Sushmita et al [14], Schoonver et al [21], Picker et al [22], and Morris et al [23]); neural network (Shadmi et al [20] and Zheng et al [24]); random forest (Sushmita et al [14] and Zheng et al [24]); support vector machine (Sushmita et al [14], Yu et al [19], and Zheng et al [24]) Population-specific: Beta geometric Erlang-2 model (Bardhan et al [25]); lasso regularization with group-level feature selection (Radovanovic [26]); logistic regression (Yu et al [19], Kelly [27], and Hasan et al [28]); multivariate logistic regression (Tabata et al [29] and Greenblatt [30]); naïve Bayes (Shameer et al [31]); tree lasso logistic regression (Jovanovic et al [32]); multivariate Cox proportional hazard model (Schmutte et al [33]); XGBoost^a^ (Morel et al [34])
Health care costs	General costs: Classification trees and clustering (Bertsimas et al [35]); linear regression (Farley et al [36], Sushmita et al [37], and Leigh et al [38]); M5 model tree (Sushmita et al [37]) High-cost patients: logistic regression (Fleishman and Cohen [39])

^a^XGBoost: eXtreme gradient boosting.

The first stream of research related to our study is on predicting readmissions. This body of literature is very large; therefore, we provide details on some representative research papers. A review paper [40] on readmission prediction models reports C statistic values between 0.55 and 0.65. Accordingly, the authors conclude that the models perform poorly. A recent study [41] reviewing articles from 2015 to 2019 reports an improvement in the C statistic values (greater than 0.75). For predicting readmissions, authors [21,42] explore the effects of physiological and medication regimens in some studies, whereas in another study [14], the authors use administrative data. Along these lines, existing studies [16,18,43] use machine learning approaches (such as deep learning and Bayesian network) to predict hospital readmission within 30 days. While using ensemble models, a model combining modified weighted boosting with a stacking algorithm shows a prediction performance 22% higher than that of a model combining the random forest algorithm, lasso algorithm, and Synthetic Minority Oversampling Technique [44]. A recent study [17] explores the use of unstructured data to predict readmission using bidirectional encoder representation from transformers. Extracting patient information from clinical notes using deep learning algorithms and then training them using graph neural networks is beneficial for prediction [45].

Next, focusing on specific subpopulation readmissions, past studies [25,29,31,46] use methods such as beta geometric Erlang-2, naïve Bayes, multivariate logistic regression, and tree-based lasso. In the case of readmissions with at least 7 past emergency department visits, boosted decision trees perform marginally better than logistic regression and the Bayes point machine [47]. A deep learning–based model built for congestive health failure patients using human-derived features, machine-derived contextual embeddings, and cost-sensitive sequential visit patterns in the EHR has the highest predictive power when compared to reduced models that use either 1 or more combinations of these [48]. eXtreme gradient boosting (XGBoost) shows better predictability than regularization techniques for predicting readmissions in mental or substance use disorders [34]. Interestingly, in a study related to psychiatric inpatients [33], the authors consider readmission within 12 months instead of the traditional 30 days to find which patient characteristics predict the time to readmission within 12 months. In terms of interpretability, existing studies [26,32] show that the tree-based lasso provides better interpretability. In an intensive care unit setting, attention-based networks may be preferable over recurrent neural networks when interpretability is of importance for a marginal decrease in accuracy [49]. Altogether, our literature review reveals that ensemble tree–based methods and deep learning approaches typically perform better than other approaches in predicting readmissions. However, none of the abovementioned studies predicts readmission charges, the focus of our study.

The literature closest to our work is on predicting health care–related costs. In one of the studies [35], the authors use classification trees and clustering algorithms to predict the general cost of health care and not specifically readmission charges. To apply these methods, the authors classify the continuous cost variable into discrete classes. In another study [50], the authors use more sophisticated machine learning methods, such as gradient boosting, an artificial neural network, and a ridge regression model, to predict cost-based classes. Although predicting general health care costs is useful, nothing can be concluded about the efficacy of these methods for predicting readmission charges because readmission is a fundamentally different phenomenon from general hospital visits. Specifically, readmission is usually associated with chronic illnesses and diseases requiring multiple visits. Moreover, bucketing a continuous variable into classes causes loss of information and may decrease predictive power.

There are studies [37,38] that predict general health care costs as a continuous variable. Apart from this, existing studies [51,52] derive costs based on predicting diagnosis-related groups (DRGs) to make operational decisions. However, as explained earlier, readmission charges are characteristically different from other types of costs. The prediction of readmission costs is considered in 1 study [14]. The authors use a limited set of methods, specifically linear regression and tree-based models, for predicting the costs (not charges). Based on our analysis of the existing literature, tree-based models and deep learning–based methods are likely to produce high prediction accuracies. We include a wide variety of prediction algorithms, including deep learning methods, to comprehensively study the problem of predicting readmission charges. Moreover, we use a data set that spans the entire United States, unlike the existing study [14] that focuses on costs (not charges) using a data set with patients from a much smaller geographic region. Thus, we can provide robust recommendations on the methods that are best suited for making readmission charge predictions across different regions of the country.

## Methods

### Data Set and its Description

We used data from the Nationwide Readmission Database (NRD) of the Healthcare Cost and Utilization Project (HCUP) by the Agency for Healthcare Research and Quality for this study [53]. The data set consists of 4 parts, namely the core data set, severity data set, hospital-level data set, and diagnosis and procedure group data set. It includes inpatient individuals from the entire United States for 2013 (first fiscal year introducing readmission policies). Readmission policies and variables in the NRD data set have not changed much after that. We used nationally representative data available publicly to find generalizable insights that can be applied to all hospitals. The total number of records in the data set was 14,325,172, including those with and without repeat hospital visits. Initially, we analyzed readmissions with respect to hospital charges using the core data set part, which consists of hospital charges for an individual. We used variables from all 4 data set parts for building predictive models (see Multimedia Appendix 1 for the categorical and numeric variables used, along with their descriptive statistics and description). After cleaning the entire data set, we identified 576,701 and 1,091,580 individuals for the 2 readmission categories, namely RSDC and RADC, respectively. Each admission record consists of the following: demographics (gender, age, median household income, etc); clinical information (diagnosis, the procedure used, etc); comorbidities (hypertension, diabetes, depression, etc); hospital details (bed size, teaching or nonteaching hospital, etc); severity details (All Patients Refined Diagnosis Related Groups for severity of illness, risk of mortality, etc); and cost-related and administrative data (length of stay, charges billed by hospitals, etc).

The data set has close to 285 mutually exclusive categories of International Classification of Diseases (ICD-9) codes for grouping diagnoses and procedures related to patients for adjusting risks. Prior studies [26] have shown that aggregated higher-level grouping of diseases was effective in providing better results than going to a specific condition at the lowest level of hierarchy in the case of pediatric readmissions. MDC codes are at a higher level than the specific DRG payment codes in this context. Per the CMS, DRGs are grouped under MDCs formed focusing on a particular medical specialty and are mutually exclusive to make them clinically consistent. They are built based on principal diagnosis codes (ICD-9 codes in this data set).

We define the terms previous admission charge and average of previous admission charges used in this study. These terms differ for RSDC and RADC. The previous admission charge for RSDC is defined as the charge billed by the hospital for only the last previous admission having the same MDC. The previous admission charge for RADC is defined as the charge billed by the hospital for the last previous admission irrespective of the MDCs. The readmission charge for RSDC and RADC is defined as the charge billed by the hospital associated with 1 readmission visit using the readmission criteria based on the definitions of RSDC and RADC, respectively. The average of previous admission charges for RSDC is defined as the average charge billed by the hospital for all the previous admissions having the same MDC. The average of previous admission charges for RADC is defined as the average charge billed by the hospital for all the previous admissions, irrespective of the MDCs.

### Ethical Considerations

We have signed the HCUP data use agreement. As per the HCUP data use agreement policy, HCUP databases are limited data sets. According to the Health Insurance Portability and Accountability Act of 1996 , review by an institutional review board is not required for limited data sets. Therefore, we did not apply for institutional review board approval for using the NRD data set [53].

### Models Used and Their Description

The average previous admission charge was considered as one of the independent variables because the previous cost proved helpful in predicting future health care costs [35,37]. All the numeric independent variables were standardized except for the average admission charge for which log transformation was applied. Log transformation was also applied to the readmission charge, namely the dependent variable. We provide brief rationales behind the models considered for RSDC and RADC below.

#### Linear Regression (Baseline Model)

It is a simple and easily interpretable method compared to other nonlinear methods. It works well when there is a linear relationship between the dependent (target) variable and independent variables. We considered using linear regression as a baseline for this study, as it has been widely used for predicting general health care costs and is also computationally efficient [36-38].

#### Lasso Regression, Elastic Net Regression, and Ridge Regression

Regularization techniques prevent overfitting and multicollinearity by constraining the loss function. We could either add the penalty as the sum of the absolute values of coefficients (*L1* penalty) in lasso or as the sum of the squared values of coefficients in the case of ridge regression (*L2* penalty). Lasso gives us sparse solutions by shrinking the estimates for some coefficients to 0, whereas ridge regression shrinks the estimates near 0. Elastic net regression takes advantage of lasso and ridge regression by linearly combining *L1* and *L2* penalties. The literature review section explains that health-related data are complex and often face multicollinearity issues. To address these challenges, we applied regularization techniques to predict readmission charges billed by hospitals. In terms of hyperparameter tuning, we tuned α (that accounts for the relative importance of the lasso and ridge regression) ranging from 0 to 1 with a step size of 0.1, and estimated λ (the regularization penalty) using cross-validation. The optimization objective in the hyperparameter tuning (in all methods used, including those introduced below) was set to minimize the root mean squared error (RMSE). We report results for lasso regression with α=1, elastic net regression with α=.5, and ridge regression with α=0.

#### XGBoost Model

It is one of the popular tree-based models for tabular data [54-56]. Prior studies [14] on predicting readmission costs (not charges) have also shown tree-based ensemble models to be beneficial. Therefore, we included this tree-based ensemble model for predicting readmission charges to take care of any nonlinearity. We chose XGBoost, as it has not been previously used in this context. Existing studies [57-59] show that a random search is sufficient and efficient in terms of the computation time for hyperparameter tuning. Hence, we performed a random search on the typical range of values for the relevant parameters depending on the type of booster [60]. The final values configured for this study are given in Table 2. The booster (type of learner) used was the tree booster (gbtree).

**Table 2:** eXtreme gradient boosting configuration details.

Configuration	Value
Number of rounds	120
Maximum depth of the tree	5
Learning rate	0.2
Subsample ratio of the training instances	0.7
L1^a^ regularization term on weights	5
L2^b^ regularization term on weights	20
Minimum loss required to make a split (gamma)	5
Subsample ratio of columns while constructing each tree	0.9

^a^L1: the sum of the absolute values of coefficients.

^b^L2: the sum of the squared values of coefficients.

#### Deep Learning Model Using Multilayer Perceptron

As discussed in the literature review section, even though deep learning–based models are more suitable for health-related data, there is no prior study that specifically predicts readmission charges using deep learning. A popular deep neural network architecture for tabular data is multilayer perceptron (MLP). Therefore, we used MLP, which requires multiple hyperparameters to be tuned. We chose the hyperparameters through a random search process, which is consistent with the recommendation provided in the literature pertaining to our case [57]. While choosing hyperparameters, we also used guidelines provided in relevant studies [61,62]. In our study, we found that models with even fewer hidden layers performed better than multiple linear regression. However, for the final configuration, we chose 4 hidden layers (beyond this, there was no further reduction in error values) to obtain fine-tuned low-error values and fewer epochs with consistent error values for the majority of the epochs. The values selected in this application are given in Table 3. The rectified linear unit was used as the activation function. The final activation function was linear, and the batch type was a minibatch.

**Table 3:** Configuration of the multilayer perceptron–based deep learning network.

Configuration	Value
Number of hidden layers	4
Number of neurons in the first hidden layer	80
Number of neurons in the second hidden layer	60
Number of neurons in the third hidden layer	50
Number of neurons in the fourth hidden layer	20
Minibatch size (weights get updated after each minibatch)	30
Momentum	0.9000
Learning rate	0.0001
Number of epochs (1 epoch = 1 forward pass + 1 backward pass)	400

### Performance Measures Used

We used 7 metrics to measure the performance of the methods. We define *n* as the total number of observations (ie, patients), *y_i_* as the actual values of readmission charges incurred by patients, 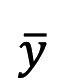 as the mean of readmission charges, and 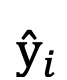 as the predicted values of readmission charges. The performance measures are provided below.

#### Mean Absolute Percentage Error

Mean absolute percentage error (MAPE) measures the error size in terms of percentage:



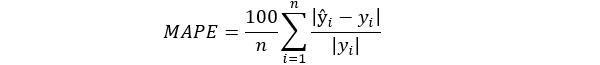



#### Root Mean Squared Error

Root mean squared error (RMSE) gives the standard deviation of the residual, which is the difference between actual and predicted values:



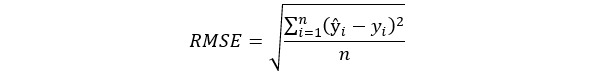



#### Mean Absolute Error

Mean absolute error (MAE) gives the average value of the errors for a given set of predictions:



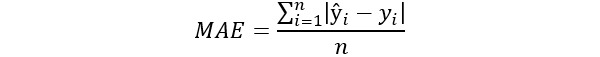



#### Root Relative Squared Error

Root relative squared error (RRSE) gives the relative comparison of what the output would have been if a naïve model (simply predicting with the mean) were used:



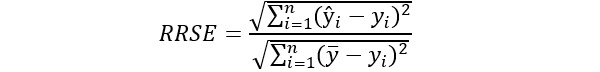



#### Relative Absolute Error

Relative absolute error (RAE) compares the total absolute error of the model to the total absolute error of the simplest model (predicting with the mean):



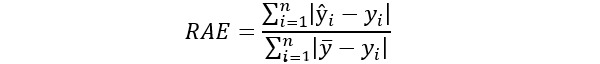



#### Normalized Root Mean Squared Error

Normalized root mean squared error (NRMSE) is used to compare models with different scales:

*NRMSE = RMSE* / 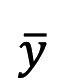



#### Mean Absolute Deviation

Mean absolute deviation (MAD) describes how the values are spread away from the mean:



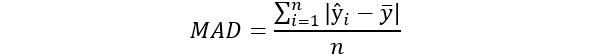



The lower the MAPE, RMSE, MAE, RAE, RRSE, NRMSE, and MAD, the better the prediction performance of the model.

## Results

**Figure 1:**
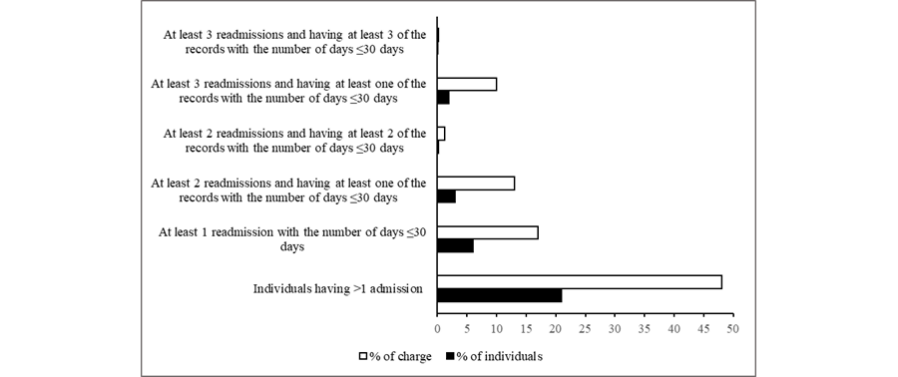
Distribution of hospital charges contributed by individuals (actual count in each category>10) for readmission with the same major diagnostic category.

**Figure 2:**
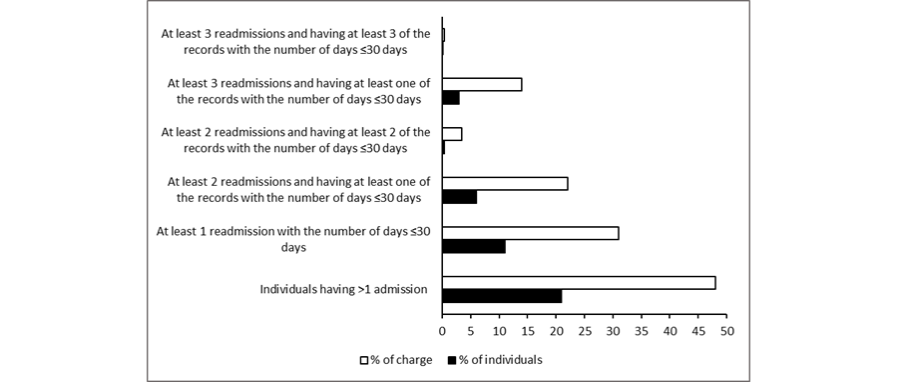
Distribution of hospital charges contributed by individuals (actual count in each category>10) for all-cause readmission category.

Initially, we analyzed the distribution of hospital charges (in percentage) contributed by individuals (in percentage) by giving different criteria for readmissions within RADC and RSDC, as shown in Figures 1 and 2. We found that 48% (US $294,802,405,683/US $614,171,678,507) of hospital charges came from 21% (2,108,143/10,038,776) of the individuals who had more than 1 admission.

Further analysis showed that the charges associated with readmissions varied from the initial admission charges for most diagnoses, with 541,090 individuals from the RADC category having readmission charges higher than the previous admission charges. Similarly, the current readmission charge was higher than the previous admission charge for 319,233 of the individuals for the RSDC category.

Next, we identified the MDCs having the highest number of readmissions for RADC and RSDC. The 2 groups are similar in terms of the MDCs with the highest number of readmissions. The categories with the highest number of readmissions for RSDC and RADC are given in Textbox 1 in descending order.

**Figure 3:**
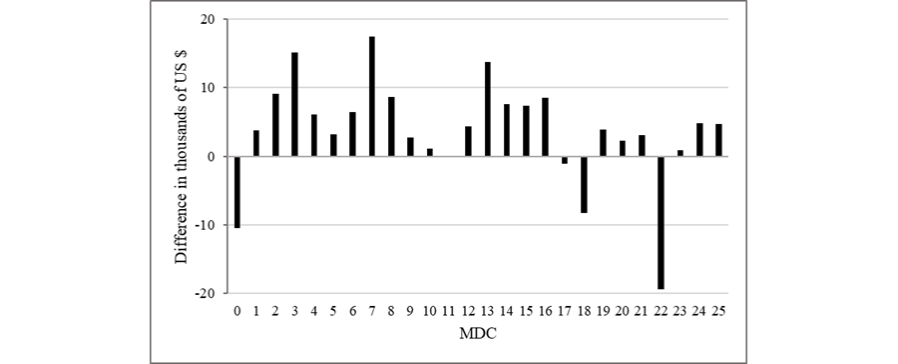
Difference between average readmission charge and average previous admission charge for readmission with the same major diagnostic category. MDC: major diagnostic category.

**Figure 4:**
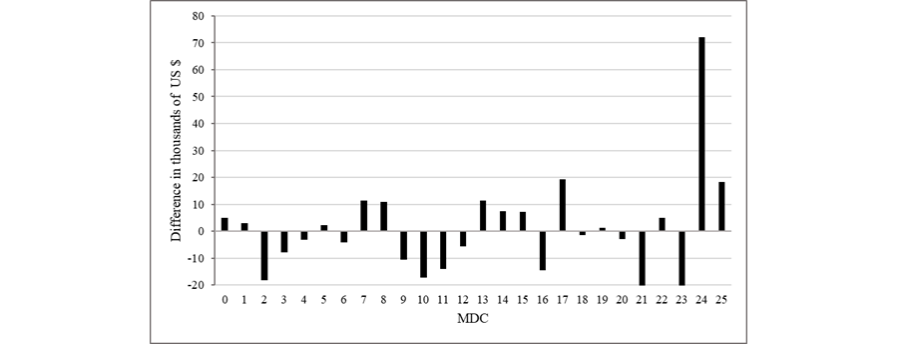
Difference between average readmission charge and average previous admission charge for all-cause readmission category. MDC: major diagnostic category.

Next, we analyzed if the average readmission charge for each MDC in RSDC and RADC varied from the previous admission charge. In Figures 3 and 4, we explain the difference between the average readmission charge (ARC) and average previous admission charge (APAC) for RSDC and RADC, respectively. In the case of RSDC, the ARC was higher than the APAC for 21 of the MDCs (Figure 3). In contrast, the ARC was higher for only 13 of the MDCs in RADC (Figure 4).

We observed that readmission charges varied from previous admission charges at the individual and aggregated levels based on the above analysis. Next, we applied various predictive methods to predict readmission charges at an individual level for RSDC and RADC. We used 10-fold cross-validation. The test results are shown in Table 4 for RSDC and Table 5 for RADC.

Tables 4 and 5 show that the deep learning–based model and XGBoost performed the best compared to all the other models for all the performance metrics in RSDC and RADC. In addition, models such as lasso, elastic net, and ridge regression using regularization techniques on a linear model showed almost the same performance. Repeated measures ANOVA revealed that the mean RMSE differed significantly across models with *P<*.001. As ANOVA is an omnibus test, we also performed a post hoc test using the Bonferroni correction method. The test showed that the mean RMSE was statistically significantly (*P*<.001) lower for the deep learning/XGBoost models when compared to that of linear regression/elastic net/lasso/ridge regression. The test showed that the mean RMSE was statistically significantly (*P<*.001) lower for the deep learning and XGBoost models when compared to that of linear regression, elastic net, lasso, and ridge regression.

**Major diagnostic categories having the highest number of readmissions listed in descending order.**

**Readmission with the same major diagnostic category**

- Diseases and disorders of the circulatory system
- Diseases and disorders of the respiratory system
- Diseases and disorders of the digestive system
- Infectious and parasitic diseases and disorders (systemic or unspecified sites)
- Diseases and disorders of the kidney and urinary tract
- Diseases and disorders of the nervous system

**All-cause readmission category**

- Diseases and disorders of the circulatory system
- Diseases and disorders of the respiratory system
- Diseases and disorders of the digestive system
- Pregnancy, childbirth, and puerperium
- Mental diseases and disorders
- Diseases and disorders of the nervous system

**Table 4:** Test results of readmission with the same major diagnostic category based on different performance measures.

Model	MAPE^a^ (%), mean (SD)	RMSE^b^, mean (SD)	MAE^c^, mean (SD)	RRSE^d^, mean (SD)	RAE^e^, mean (SD)	NRMSE^f^, mean (SD)	MAD^g^, mean (SD)
Linear regression	4.268 (0.035)	0.564 (0.002)	0.431 (0.002)	0.546 (0.005)	0.528 (0.004)	0.055 (0.000)	0.042 (0.000)
Lasso	4.269 (0.036)	0.564 (0.002)	0.431 (0.002)	0.546 (0.005)	0.528 (0.004)	0.055 (0.000)	0.042 (0.000)
Elastic net	4.269 (0.036)	0.564 (0.002)	0.431 (0.002)	0.546 (0.005)	0.528 (0.004)	0.055 (0.000)	0.042 (0.000)
Ridge	4.299 (0.037)	0.565 (0.003)	0.434 (0.002)	0.547 (0.005)	0.531 (0.004)	0.055 (0.000)	0.042 (0.001)
XGBoost^h^	3.171 (0.027)	0.421 (0.003)	0.321 (0.002)	0.407 (0.004)	0.393 (0.003)	0.041 (0.001)	0.031 (0.000)
Deep learning	3.202 (0.022)	0.427 (0.003)	0.326 (0.002)	0.413 (0.004)	0.399 (0.003)	0.041 (0.001)	0.032 (0.000)

^a^MAPE: mean absolute percentage error.

^b^RMSE: root mean squared error.

^c^MAE: mean absolute error.

^d^RRSE: root relative squared error.

^e^RAE: relative absolute error.

^f^NRMSE: normalized root mean squared error.

^g^MAD: mean absolute deviation.

^h^XGBoost: eXtreme gradient boosting.

**Table 5:** Test results of all-cause readmission category based on different performance measures.

Model	MAPE^a^ (%), mean (SD)	RMSE^b^, mean (SD)	MAE^c^, mean (SD)	RRSE^d^, mean (SD)	RAE^e^, mean (SD)	NRMSE^f^, mean (SD)	MAD^g^, mean (SD)
Linear regression	4.208 (0.047)	0.558 (0.004)	0.427 (0.003)	0.554 (0.005)	0.537 (0.005)	0.054 (0.000)	0.041 (0.001)
Lasso	4.208 (0.047)	0.558 (0.004)	0.427 (0.003)	0.554 (0.005)	0.537 (0.005)	0.054 (0.000)	0.041 (0.001)
Elastic net	4.209 (0.047)	0.558 (0.004)	0.427 (0.003)	0.554 (0.005)	0.537 (0.005)	0.054 (0.000)	0.041 (0.001)
Ridge	4.240 (0.049)	0.559 (0.005)	0.429 (0.003)	0.555 (0.005)	0.531 (0.005)	0.054 (0.000)	0.042 (0.001)
XGBoost^h^	3.121 (0.019)	0.414 (0.002)	0.317 (0.002)	0.410 (0.001)	0.399 (0.002)	0.040 (0.000)	0.031 (0.000)
Deep learning	3.103 (0.018)	0.413 (0.003)	0.316 (0.003)	0.410 (0.002)	0.397 (0.003)	0.040 (0.000)	0.031 (0.000)

^a^MAPE: mean absolute percentage error.

^b^RMSE: root mean squared error.

^c^MAE: mean absolute error.

^d^RRSE: root relative squared error.

^e^RAE: relative absolute error.

^f^NRMSE: normalized root mean squared error.

^g^MAD: mean absolute deviation.

^h^XGBoost: eXtreme gradient boosting.

## Discussion

### Principal Results and Comparison With Prior Work

This study shows that national administrative data can be used to build effective predictive models for hospital charges billed for readmissions, even if there are different criteria for readmissions. The deep learning–based algorithm and XGBoost outperformed all other algorithms. Based on our experiments, we also made a few observations specific to configuring XGBoost. While configuring the XGBoost model, we found that using the gradient descent of the tree-type booster gave the best performance compared to other boosters such as a linear booster or dropouts with multiple additive regression tree boosters. Moreover, in the same context, setting the booster to linear with regularization for XGBoost gave a performance equivalent to linear, lasso, elastic net, and ridge regression.

In summary, this study makes 2 important contributions. To the best of our knowledge, this is the first study to apply regularization techniques, a tree-based ensemble model using XGBoost, and deep learning–based models for predicting readmission charges billed by hospitals. Deep learning–based models and XGBoost have proven useful in modeling health-related data. A related study that focused on predicting readmission costs (not charges) used only linear regression and tree-based models on narrow data sets (~10k samples) with limited features, and hence, its applicability in different geographies is questionable. Besides, it predicted readmission costs (not charges) using only the all-cause definition of readmission. Our study considered readmission using MDCs instead of DRGs by using different MDC criteria to determine which models would be suitable for predicting readmission charges.

### Implications

This study has 2 practical implications. First, health systems use high-risk care management programs to improve health outcomes in individuals with complex needs and reduce costs. As these programs are resource-intensive and expensive, health systems use costs as a proxy to identify individuals suitable for these programs [63]. Our study related to readmissions will aid such programs by prescribing models that will provide reliable estimates of readmission charges.

Second, hospital reimbursement mainly depends on DRG codes and the case mix index (CMI). The CMI is calculated as the average DRG weight of the hospitals’ inpatient discharges. A higher CMI would indicate more reimbursement for hospitals. As the CMI is not directly tied to either hospital charges (which can vary depending on various factors specific to the hospital, such as staffing expenses and technologies used) or individual-specific expenses, hospitals often do not get reimbursed for the services they have provided [64]. In this study, we predicted readmission charges that will give hospitals a better estimate of the cost they are going to incur in case the patients get readmitted. Now, hospitals can use the CMI and DRGs to determine their reimbursement amounts and compare that with the estimated charges. If there are any differences in the amount, hospitals can now more effectively plan for mitigation strategies. Thus, in a nutshell, our study can be helpful for health care policymakers and hospital planners.

### Limitations and Future Research

Modeling readmission likelihood and the length of stay are also crucial in readmissions, as these outcomes influence one another. Moreover, modeling readmission charges, readmission likelihood, and length of stay might be more beneficial than focusing only on modeling readmission charges. In this study, we identified readmissions belonging to RSDC and RADC. We will also use the term readmission in the readmission with different major diagnostic category (RDDC) for our future analysis. RDDC will consider readmission as an admission to a hospital within 30 days of discharge from the same or another hospital with the cause of readmission being different. We will then build predictive models for RDDC. Then, we will compare the predictive models built for RDDC with those built for RSDC.

In this study, we considered the standard defined categories of MDCs as the cause of readmission. The standard defined categories of MDCs belong to either a single organ system or an etiology. For our future study, we will consider correlated categories in terms of the set of related health complications that eventually lead to readmissions. These categories may span multiple MDCs. We expect that such recategorizations could help in the better prediction of charges. The recategorization in terms of correlated categories would significantly contribute to health care economics.

### Conclusions

Readmissions are one of the main contributors to health care costs. However, most previous studies have focused mainly on predicting early readmissions. The implementation of the Hospital Readmissions Reduction Program has mixed reviews, with no conclusion regarding its effectiveness. This study aimed to determine if readmission charges, which vary from initial admission charges, could be accurately predicted. Results revealed that the deep learning–based model and XGBoost performed the best in terms of all performance measures. MDCs can be used to accurately predict charges billed by hospitals for readmissions.

## Appendix

Multimedia Appendix 1 Variables used in this study, along with their descriptions and descriptive statistics.

## Abbreviations

APAC: average previous admission charge
ARC: average readmission charge
CMI: case mix index
CMS: Centers for Medicare and Medicaid Services
DRG: diagnosis-related group
EHR: electronic health record
HCUP: Healthcare Cost and Utilization Project
ICD: International Classification of Diseases
MAD: mean absolute deviation
MAE: mean absolute error
MAPE: mean absolute percentage error
MDC: major diagnostic category
MLP: multilayer perceptron
NRD: Nationwide Readmission Database
NRMSE: normalized root mean squared error
RADC: all-cause readmission category
RAE: relative absolute error
RDDC: readmission with different major diagnostic category
RMSE: root mean squared error
RRSE: root relative squared error
RSDC: readmission with the same major diagnostic category
XGBoost: eXtreme gradient boosting

## References

[1] Jamoom E, Yang N (2016). Table of electronic health record adoption use among office-based physicians in the US by state: 2015 National Electronic Health Records Survey. National Electronic Health Records Survey: 2015 State and National Electronic Health Record Adoption Summary Tables.

[2] Atherton J (2011). Development of the electronic health record. AMA J Ethics.

[3] OECD (2019). Health at a Glance 2019: OECD Indicators.

[4] Hines AL, Barrett ML, Jiang HJ, Steinar CA (2014). Conditions with the largest number of adult hospital readmissions by payer, 2011. Healthcare Cost and Utilization Project (HCUP) Statistical Briefs. Brief #172.

[5] Wilson L (2019). MA patients' readmission rates higher than traditional Medicare, study finds; 2019. HEALTHCAREDIVE.

[6] LaPointe J (2020). CDC: 1 in 11 COVID-19 inpatients experience a hospital readmission. Xtelligent Healthcare Media.

[7] Vaid A, Jaladanki S, Xu J, Teng S, Kumar A, Lee S, Somani S, Paranjpe I, De Freitas JK, Wanyan T, Johnson K, Bicak M, Klang E, Kwon Y, Costa A, Zhao S, Miotto R, Charney A, Böttinger E, Fayad Z, Nadkarni G, Wang F, Glicksberg BS (2021). Federated learning of electronic health records to improve mortality prediction in hospitalized patients with COVID-19: machine learning approach. JMIR Med Inform.

[8] Tran L, Chi L, Bonti A, Abdelrazek M, Phoebe Chen Y-P (2021). Mortality prediction of patients with cardiovascular disease using medical claims data under artificial intelligence architectures: validation study. JMIR Med Inform.

[9] Zhao P, Yoo I, Naqvi SH (2021). Early prediction of unplanned 30-day hospital readmission: model development and retrospective data analysis. JMIR Med Inform.

[10] Conway A, Jungquist CR, Chang K, Kamboj N, Sutherland J, Mafeld S, Parotto M (2021). Predicting prolonged apnea during nurse-administered procedural sedation: machine learning study. JMIR Perioper Med.

[11] Hou C, Zhong X, He P, Xu B, Diao S, Yi F, Zheng H, Li J (2020). Predicting breast cancer in Chinese women using machine learning techniques: algorithm development. JMIR Med Inform.

[12] Lee E, Jung SY, Hwang HJ, Jung J (2021). Patient-level cancer prediction models from a nationwide patient cohort: model development and validation. JMIR Med Inform.

[13] (2007). Payment policy for inpatient readmissions promoting greater efficiency in Medicare. MedPAC.

[14] Sushmita S, Khulbe G, Hasan A, Newman S, Ravindra P, Roy SB, De Cock M, Teredesai A (2016). Predicting 30-day risk and cost of “all-cause” hospital readmission. The Workshops of the Thirtieth AAAI Conference on Artificial Intelligence Expanding the Boundaries of Health Informatics Using AI: Technical Report WS-16-08.

[15] Jamei M, Nisnevich A, Wetchler E, Sudat S, Liu E (2017). Predicting all-cause risk of 30-day hospital readmission using artificial neural networks. PLoS ONE.

[16] Cai X, Perez-Concha O, Coiera E, Martin-Sanchez F, Day R, Roffe D, Gallego B (2016). Real-time prediction of mortality, readmission, and length of stay using electronic health record data. J Am Med Inform Assoc.

[17] Huang K, Altosaar J, Ranganathan R ClinicalBERT: modeling clinical notes and predicting hospital readmission. ArXiv.

[18] Wang H, Cui Z, Chen Y, Avidan M, Abdallah AB, Kronzer A (2018). Predicting hospital readmission via cost-sensitive deep learning. IEEE/ACM Trans Comput Biol Bioinform.

[19] Yu S, Farooq F, van Esbroeck A, Fung G, Anand V, Krishnapuram B (2015). Predicting readmission risk with institution-specific prediction models. Artif Intell Med.

[20] Shadmi E, Flaks-Manov N, Hoshen M, Goldman O, Bitterman H, Balicer RD (2015). Predicting 30-day readmissions with preadmission electronic health record data. Med Care.

[21] Schoonover H, Corbett CF, Weeks DL, Willson MN, Setter SM (2014). Predicting potential postdischarge adverse drug events and 30-day unplanned hospital readmissions from medication regimen complexity. J Patient Saf.

[22] Picker D, Heard K, Bailey TC, Martin NR, LaRossa GN, Kollef MH (2015). The number of discharge medications predicts thirty-day hospital readmission: a cohort study. BMC Health Serv Res.

[23] Morris PE, Griffin L, Berry M, Thompson C, Hite RD, Winkelman C, Hopkins RO, Ross A, Dixon L, Leach S, Haponik E (2011). Receiving early mobility during an intensive care unit admission is a predictor of improved outcomes in acute respiratory failure. Am J Med Sci.

[24] Zheng B, Zhang J, Yoon SW, Lam SS, Khasawneh M, Poranki S (2015). Predictive modeling of hospital readmissions using metaheuristics and data mining. Expert Syst Appl.

[25] Bardhan I, Jeong-ha (C), Oh Z (E), Kirk, Kirksey Z (2015). Predictive analytics for readmission of patients with congestive heart failure. Inf Syst Res.

[26] Radovanovic S, Vukicevic M, Kovacevic A, Stiglic G, Obradovic Z, Holmes J, Bellazzi R, Sacchi L, Peek N (2015). Domain knowledge based hierarchical feature selection for 30-day hospital readmission prediction. Artificial Intelligence in Medicine. AIME 2015. Lecture Notes in Computer Science(), vol 9105.

[27] Kelly M, Sharp Linda, Dwane Fiona, Kelleher Tracy, Comber Harry (2012). Factors predicting hospital length-of-stay and readmission after colorectal resection: a population-based study of elective and emergency admissions. BMC Health Serv Res.

[28] Hasan O, Meltzer DO, Shaykevich SA, Bell CM, Kaboli PJ, Auerbach AD, Wetterneck TB, Arora VM, Zhang J, Schnipper JL (2010). Hospital readmission in general medicine patients: a prediction model. J Gen Intern Med.

[29] Tabata M, Shimizu R, Kamekawa D, Kato M, Kamiya K, Akiyama A, Kamada Y, Tanaka S, Noda C, Masuda T (2014). Six-minute walk distance is an independent predictor of hospital readmission in patients with chronic heart failure. Int Heart J.

[30] Greenblatt DY, Weber SM, O'Connor ES, LoConte NK, Liou J, Smith MA (2010). Readmission after colectomy for cancer predicts one-year mortality. Ann Surg.

[31] Shameer K, Johnson KW, Yahi A, Miotto R, Li LI, Ricks D, Jebakaran J, Kovatch P, Sengupta PP, Gelijns S, Moskovitz A, Darrow B, David DL, Kasarskis A, Tatonetti NP, Pinney S, Dudley JT (2017). Predictive modeling of hospital readmission rates using electronic medical record-wide machine learning: a case-study using Mount Sinai heart failure cohort. Pac Symp Biocomput.

[32] Jovanovic M, Radovanovic S, Vukicevic M, Van Poucke S, Delibasic B (2016). Building interpretable predictive models for pediatric hospital readmission using tree-lasso logistic regression. Artif Intell Med.

[33] Schmutte T, Dunn CL, Sledge WH (2010). Predicting time to readmission in patients with recent histories of recurrent psychiatric hospitalization: a matched-control survival analysis. J Nerv Ment Dis.

[34] Morel D, Yu KC, Liu-Ferrara A, Caceres-Suriel AJ, Kurtz SG, Tabak YP (2020). Predicting hospital readmission in patients with mental or substance use disorders: a machine learning approach. Int J Med Inform.

[35] Bertsimas D, Bjarnadóttir MV, Kane MA, Kryder JC, Pandey R, Vempala S, Wang G (2008). Algorithmic prediction of health-care costs. Oper Res.

[36] Farley JF, Harley CR, Devine JW (2006). A comparison of comorbidity measurements to predict healthcare expenditures. Am J Manag Care.

[37] Sushmita S, Newman S, Marquardt J, Ram P, Prasad V, Cock MD, Teredesai A (2015). Population cost prediction on public healthcare datasets. DH '15: Proceedings of the 5th International Conference on Digital Health 2015.

[38] Leigh JP, Hubert HB, Romano PS (2005). Lifestyle risk factors predict healthcare costs in an aging cohort. Am J Prev Med.

[39] Fleishman JA, Cohen JW (2010). Using information on clinical conditions to predict high-cost patients. Health Serv Res.

[40] Kansagara D, Englander H, Salanitro A, Kagen D, Theobald C, Freeman M, Kripalani S (2011). Risk prediction models for hospital readmission: a systematic review. JAMA.

[41] Mahmoudi E, Kamdar N, Kim N, Gonzales G, Singh K, Waljee AK (2020). Use of electronic medical records in development and validation of risk prediction models of hospital readmission: systematic review. BMJ.

[42] Xue Y, Klabjan D, Luo Y (2019). Predicting ICU readmission using grouped physiological and medication trends. Artif Intell Med.

[43] Xiao C, Ma T, Dieng AB, Blei DM, Wang F (2018). Readmission prediction via deep contextual embedding of clinical concepts. PLoS One.

[44] Yu K, Xie X (2020). Predicting hospital readmission: a joint ensemble-learning model. IEEE J Biomed Health Inform.

[45] Golmaei SN, Luo X (2021). DeepNote-GNN: predicting hospital readmission using clinical notes and patient network. Proceedings of the 12th ACM Conference on Bioinformatics, Computational Biology, and Health Informatics.

[46] Cui S, Wang D, Wang Y, Yu P, Jin Y (2018). An improved support vector machine-based diabetic readmission prediction. Comput Methods Programs Biomed.

[47] Ben-Assuli O, Padman R (2018). Analysing repeated hospital readmissions using data mining techniques. Health Syst (Basingstoke).

[48] Ashfaq A, Sant'Anna A, Lingman M, Nowaczyk S (2019). Readmission prediction using deep learning on electronic health records. J Biomed Inform.

[49] Barbieri S, Kemp J, Perez-Concha O, Kotwal S, Gallagher M, Ritchie A, Jorm L (2020). Benchmarking deep learning architectures for predicting readmission to the ICU and describing patients-at-risk. Sci Rep.

[50] Morid MA, Kawamoto K, Ault T, Dorius J, Abdelrahman S (2017). Supervised learning methods for predicting healthcare costs: systematic literature review and empirical evaluation. AMIA Annu Symp Proc.

[51] Liu J, Capurro D, Nguyen A, Verspoor K (2021). Early prediction of diagnostic-related groups and estimation of hospital cost by processing clinical notes. NPJ Digit Med.

[52] Gartner D, Kolisch R, Neill DB, Padman R (2015). Machine learning approaches for early DRG classification and resource allocation. INFORMS J Comput.

[53] HCUP Nationwide Readmissions Database (NRD) (2013). Healthcare Cost Utilization Project (HCUP). Agency for Healthcare Research Quality.

[54] Shwartz-Ziv R, Armon A (2022). Tabular data: deep learning is not all you need. Inf Fusion.

[55] Edgari E, Thiojaya J, Qomariyah NN (2022). The impact of Twitter sentiment analysis on bitcoin price during COVID-19 with XGBoost.

[56] Sahin EK (2020). Assessing the predictive capability of ensemble tree methods for landslide susceptibility mapping using XGBoost, gradient boosting machine, and random forest. SN Appl Sci.

[57] Bergstra J, Bengio Y (2012). Random search for hyper-parameter optimization. J Mach Learn Res.

[58] Mantovani RG, Rossi AL, Vanschoren J, Bischl B, De Carvalho AC (2015). Effectiveness of random search in SVM hyper-parameter tuning.

[59] Park J, Lee Y, Lee J (2021). Assessment of machine learning algorithms for land cover classification using remotely sensed data. Sens Mater.

[60] Wade C (2020). Hands-On Gradient Boosting With XGBoost and scikit-learn: Perform Accessible Machine Learning and Extreme Gradient Boosting With Python.

[61] Bengio Y (2012). Practical recommendations for gradient-based training for deep architectures. Neural networks: Tricks of the trade.

[62] Sheela K, Deepa SN (2013). Review on methods to fix number of hidden neurons in neural networks. Math Probl Eng.

[63] Obermeyer Z, Powers B, Vogeli C, Mullainathan S (2019). Dissecting racial bias in an algorithm used to manage the health of populations. Science.

[64] Ericson C (2021). Is case mix index still a relevant key performance indicator?. Journal of AHIMA.

