# Supplementary material for: Predicting Readmission Charges Billed by Hospitals: Machine Learning Approach
Source: JMIR Med Inform. 2022 Aug 30;10(8):e37578. doi: 10.2196/37578 (PMC9472041; doi:10.2196/37578)
Supplement: Multimedia Appendix 1 [file medinform_v10i8e37578_app1.pdf]

Multimedia Appendix 1

Variables used in this study, along with their descriptions and descriptive statistics

(Variable names and their explanations retrieved from the AHRQ website) [53]

Table S1 Variable descriptions and descriptive statistics

Readmission Category			RSDC	RADC
Variables and their description			Descriptive statistics	
Continuous Variables			Mean (prior normalization)	
Age		Patient's age (in years)	56	60
Discharge weight		Used for calculating national estimates	2.38	2.36
Length of stay		Patient's Length of stay	6	6
Chronic Condition		Patient's number of chronic conditions	5	5
Diagnoses		Number of diagnoses coded	11	12
Procedures		Number of procedures coded	1	1
Injury		Number of external causes of injury code	0	0
Hospital readmission charge		Costs billed by the hospital for the readmission	$52715	$53041
Previous charge		Costs billed by the hospital for the last previous admission	$48004	$54925
Average previous charge		Average of costs billed by hospitals for all previous admissions	$48273	$55774
Hospital's discharge		Total number of discharges for each hospital	19806	20311
Categorical Variables			Percentage (The highest category type is included in parentheses)	
Variable Indicator	Category			
	Type	Description		
	0	No	78.72	77.79

Weekend admission	1	Yes	(0)	(0)
Patient disposition	1	Routine	59.75 (1)	51.01 (1)
	2	Transfer to short term hospital		
	5	Other transfers, including skilled nursing facility, intermediate care, and another type of facility		
	6	Home health care		
	7	Against medical advice		
	20	Died in hospital		
	99	Discharged alive, unknown destination		
Quarter Year	1	January to March	28.66 (4)	29.58 (4)
	2	April to June		
	3	July to September		
	4	October to December		
Elective Admission	0	No	83.61 (0)	87.41 (0)
	1	Yes		
Sex	0	Male	53.74 (1)	53.45 (1)
	1	Female		
Emergency department (ED) services	0	Record does not meet any HCUP ED criteria	39.76 (1)	43.6 (1)
	1	ED revenue code was on State Inpatient Database (SID) record		
	2	ED charge reported on SID record		
	3	ED CPT procedure code on SID record		
	4	Other indication of ED services		
MDC	0	Pre-MDC	20.35 (5)	16.83 (5)
	1	Diseases and disorders of the nervous System		
	2	Diseases and disorders of the eye		
	3	Diseases and disorders of the ear, nose, mouth, and throat		
	4	Diseases and disorders of the respiratory system		
	5	Diseases and disorders of the circulatory system		
	6	Diseases and disorders of the digestive system		
	7	Diseases and disorders of the hepatobiliary system and pancreas		

	8	Diseases and disorders of the musculoskeletal system and connective tissue		
	9	Diseases and disorders of the skin, subcutaneous tissue, and breast		
	10	Diseases and disorders of the endocrine, nutritional and metabolic system		
	11	Diseases and disorders of the kidney and urinary tract		
	12	Diseases and disorders of the male reproductive system		
	13	Diseases and disorders of the female reproductive system		
	14	Pregnancy, childbirth, and puerperium		
	15	Newborn and other neonates (Perinatal period)		
	16	Diseases and disorders of the blood and blood forming organs and immunological disorders		
	17	Myeloproliferative diseases and disorders (Poorly differentiated neoplasms)		
	18	Infectious and parasitic diseases and disorders (Systemic or unspecified sites)		
	19	Mental diseases and disorders		
	20	Alcohol/Drug use or induced mental disorders		
	21	Injuries, poison and toxic effect of drugs		
	22	Burns		
	23	Factors influencing health status and other contacts with health services		
	24	Multiple significant trauma		
	25	Human immunodeficiency virus infection		
Stratum	There are 89 randomly assigned categories of NRD stratum based on hospital-related factors.		7.70 (1)	7.18 (1)
Operating procedure on discharge record	0	No	77.80 (0)	81.14 (0)
	1	Yes		

Insurance type	1	Medicare	49.03 (1)	56.16 (1)
	2	Medicaid		
	3	Private insurance		
	4	Uninsured (self-pay)		
	5	Uninsured (no charge)		
	6	Other		
Patient's location using based on the National Center for Health Statistics (NCHS) urban-rural classification scheme	1	"Central" counties of metro areas of >=1 million population	32.59 (1)	31.63 (1)
	2	"Fringe" counties of metro areas of >=1 million population		
	3	Counties in metro areas of 250,000–999,999 population		
	4	Counties in metro areas of 50,000–249,999 population		
	5	Micropolitan counties		
	6	Not metropolitan or micropolitan counties		
The location patient had been transferred: rehabilitation, evaluation, or other aftercare	0	No	99.08 (0)	98.95 (0)
	1	Yes		
Patient's residency status	0	No	96.65 (1)	96.69 (1)
	1	Yes		
Patient's same day event status	0	Not a combined transfer or other same day stay records	96.48 (0)	0-96.21 (0)
	1	Combined transfer involving two discharges from different hospitals		
	2	Combined same day stay involving two discharges at different hospitals		
	3	Combined same day stay involving two discharges at the same hospital		
	4	Combined same-day stays involving three or more discharges at same or different hospitals		
Median household income quartiles based	1	$1 - $37,999	30.70 (1)	30.08 (1)
	2	$38,000 - $47,999		
	3	$48,000 - $63,999		

on patient's zip code	4	$64,000 or more.		
Size of hospital with respect to number of beds	1	Small	67.98	67.23
	2	Medium		
	3	Large		
Hospital's teaching status	0	Metropolitan non-teaching	53.20 (1)	51.05 (1)
	1	Metropolitan teaching		
	2	Non-metropolitan		
Hospital's location	1	Large metropolitan areas with at least 1 million residents	59.01 (1)	58.00 (1)
	2	Small metropolitan areas with less than 1 million residents		
	3	Micropolitan areas		
	4	Not metropolitan or micropolitan		
Hospital's ownership	1	Government, nonfederal [public]	68.51 (2)	68.60 (2)
	2	Private, not-for-profit [voluntary]		
	3	Private, investor-owned [proprietary]		
3M APR-DRG: Risk of mortality subclass	0	No class specified	42.83 (1)	34.42 (1)
	1	Minor likelihood of dying		
	2	Moderate likelihood of dying		
	3	Major likelihood of dying		
	4	Extreme likelihood of dying		
3M APR-DRG: Severity of illness subclass	0	No class specified	40.83 (2)	37.48 (2)
	1	Minor loss of function (includes cases with no comorbidity or complications)		
	2	Moderate loss of function		
	3	Major loss of function		
	4	Extreme loss of function		
Acquired immune deficiency syndrome comorbidity	0	No	99.75 (0)	99.73 (0)
	1	Yes		
Alcohol use comorbidity	0	No	94.75 (0)	94.97 (0)
	1	Yes		

Anemia deficiency comorbidity	0	No	77.94 (0)	74.98 (0)
	1	Yes		
Rheumatoid arthritis/ collagen vascular disease comorbidity	0	No	97.29 (0)	96.85 (0)
	1	Yes		
Blood loss anemia comorbidity	0	No	97.44 (0)	97.91 (0)
	1	Yes		
Congestive heart failure comorbidity	0	No	90.40 (0)	85.99 (0)
	1	Yes		
Chronic pulmonary disease comorbidity	0	No	78.49 (0)	76.87 (0)
	1	Yes		
Coagulopathy comorbidity	0	No	94.11 (0)	93.37 (0)
	1	Yes		
Depression comorbidity	0	No	88.79 (0)	88.01 (0)
	1	Yes		
Uncomplicated diabetes comorbidity	0	No	79.36 (0)	77.65 (0)
	1	Yes		
Diabetes with chronic complications comorbidity	0	No	94.86 (0)	93.81 (0)
	1	Yes		
Drug abuse comorbidity	0	No	93.73 (0)	94.75 (0)
	1	Yes		
Hypertension comorbidity	0	No	50.48 (0)	45.63 (0)
	1	Yes		
Hypothyroidism comorbidity	0	No	88.77 (0)	87.35 (0)
	1	Yes		
	0	No		

Liver disease comorbidity	1	Yes	96.49 (0)	95.78 (0)
Lymphoma comorbidity	0	No	99.07 (0)	98.60 (0)
	1	Yes		
Fluid and electrolyte disorder comorbidity	0	No	72.95 (0)	68.07 (0)
	1	Yes		
Metastatic cancer comorbidity	0	No	96.89 (0)	95.77 (0)
	1	Yes		
Neurological disorders comorbidity	0	No	92.01 (0)	90.05 (0)
	1	Yes		
Obesity comorbidity	0	No	88.39 (0)	88.39 (0)
	1	Yes		
Paralysis comorbidity	0	No	97.20 (0)	96.44 (0)
	1	Yes		
Peripheral vascular disorders comorbidity	0	No	93.51 (0)	92.53 (0)
	1	Yes		
Psychosis comorbidity	0	No	94.68 (0)	94.17 (0)
	1	Yes		
Pulmonary circulation disorders comorbidity	0	No	97.40 (0)	96.35 (0)
	1	Yes		
Renal Failure comorbidity	0	No	84.06 (0)	81.39 (0)
	1	Yes		
Solid tumor without metastasis comorbidity	0	No	97.21 (0)	96.38 (0)
	1	Yes		
Peptic ulcer disease	0	No	99.95 (0)	99.95 (0)

excluding bleeding comorbidity	1	Yes		
Valvular disease comorbidity	0	No	96.68 (0)	95.17 (0)
	1	Yes		
Weight loss comorbidity	0	No	92.87 (0)	91.40 (0)
	1	Yes		
